# Agroecosystem energy transitions in the old and new worlds: trajectories and determinants at the regional scale

**DOI:** 10.1007/s10113-017-1261-y

**Published:** 2017-12-07

**Authors:** Simone Gingrich, Inés Marco, Eduardo Aguilera, Roc Padró, Claudio Cattaneo, Geoff Cunfer, Gloria I. Guzmán, Joshua MacFadyen, Andrew Watson

**Affiliations:** 1 Institute of Social Ecology, Alpen-Adria Universitaet Klagenfurt, Klagenfurt, Austria; 2 Department of Economic History, Institutions, Policy and World Economy, Universitat de Barcelona, Barcelona, Spain; 3 Agroecosystems History Laboratory, Universidad Pablo de Olavide, Sevilla, Spain; 4 Barcelona Institute of Regional and Metropolitan Studies, Universitat Autonoma de Barcelona, Barcelona, Spain; 5 Department of History, University of Saskatchewan, Saskatoon, Canada; 6 School of Historical Philosophical and Religious Studies and School of Sustainability, Arizona State University, Tempe, AZ USA

**Keywords:** Agroecosystem energy transition, Long-term socio-ecological research, Energy return on investment, Energy efficiency

## Abstract

Energy efficiency in biomass production is a major challenge for a future transition to sustainable food and energy provision. This study uses methodologically consistent data on agroecosystem energy flows and different metrics of energetic efficiency from seven regional case studies in North America (USA and Canada) and Europe (Spain and Austria) to investigate energy transitions in Western agroecosystems from the late nineteenth to the late twentieth centuries. We quantify indicators such as external final energy return on investment (EFEROI, i.e., final produce per unit of external energy input), internal final EROI (IFEROI, final produce per unit of biomass reused locally), and final EROI (FEROI, final produce per unit of total inputs consumed). The transition is characterized by increasing final produce accompanied by increasing external energy inputs and stable local biomass reused. External inputs did not replace internal biomass reinvestments, but added to them. The results were declining EFEROI, stable or increasing IFEROI, and diverging trends in FEROI. The factors shaping agroecosystem energy profiles changed in the course of the transition: Under advanced organic and frontier agriculture of the late nineteenth and early twentieth centuries, population density and biogeographic conditions explained both agroecosystem productivity and energy inputs. In industrialized agroecosystems, biogeographic conditions and specific socio-economic factors influenced trends towards increased agroecosystem specialization. The share of livestock products in a region’s final produce was the most important factor determining energy returns on investment.

**Electronic supplementary material:**

The online version of this article (10.1007/s10113-017-1261-y) contains supplementary material, which is available to authorized users.

## Introduction

The notion of energy transition describes the shift from traditional energy carriers (notably biomass) to modern energy sources, in particular fossil fuels (Grübler 2008). This process did not happen simultaneously around the globe or even within world regions (Gales et al. 2007). Still, consensus holds that industrialization entailed an overlapping succession of coal (“first industrial revolution”) followed by crude oil and later natural gas and other modern energy sources (“second and third industrial revolutions”) as major energy suppliers to socio-economic activities (Kander et al. 2014). The concept of energy transitions is particularly useful from the perspective of a biophysically informed economic history, in which technical energy use is closely connected to economic growth (Ayres and Warr 2010).

From a socio-ecological perspective interested in the interplay of socio-economic and ecological processes, it is worthwhile to extend the analysis beyond technical energy use to all energy carriers used in a society, including biomass used as food, feed, fibers, and construction materials. The concept of social metabolism (Fischer-Kowalski 1998; Gonzalez de Molina and Toledo 2014) has been used in long-term socio-ecological research (Haberl et al. 2006) to quantify changes in socio-economic material and energy use over time. This approach has demonstrated that modern energy carriers did not substitute biomass as major energy input to society, but were used in addition to increasing amounts of biomass (Krausmann 2001; Kuskova et al. 2008; Soto et al. 2016). The changing relationship between material and energy use on the one hand and land use on the other has been described as a “socio-ecological transition” (Fischer-Kowalski and Haberl 2007; Krausmann et al. 2008). However, long-term socio-ecological research has thus far largely focused on the extraction of biomass and energy from the environment, and knowledge gaps exist with regard to how much and which type of energy was used in the process of biomass extraction.

The ratio of energy outputs to inputs, or energy return on investment (EROI), is a concept suitable to shed light on this issue. It was first developed in a study on migrating fish (Hall 1972) and later applied in the investigation of the energy efficiency of fossil energy generation (Cleveland et al. 1984; Murphy and Hall 2011; Guilford et al. 2011; Court and Fizaine 2017). In parallel, the same concept was applied to agricultural systems (Pimentel et al. 1973; Leach 1976). Currently, the literature on agricultural EROIs features three major strands:

1. A limited, but relevant, amount of studies investigates energy returns on investment at the national scale. The energy efficiency of agricultural sectors is compared over a period of several decades up to a century (Cleveland 1995; Ozkan et al. 2004; Hamilton et al. 2013) and in some cases, it covers the whole agro-food system (e.g., Steinhart and Steinhart 1974). These studies yield different results. Both energy input and agricultural production increased in most cases in the long run, resulting in more fossil fuel input per unit of final agricultural product in the course of industrialization (Pimentel et al. 1973, Smil 2000). In recent decades, however, the energy return on investment increased in some countries (e.g., the USA) (Hamilton et al. 2013), declined in others (e.g., Turkey) (Ozkan et al. 2004), or remained stable (e.g., Canada) (Hamilton et al. 2013).
2. Some national-scale analyses focus on energy efficiency at the level of individual crops or agricultural products, either over time (Smil et al. 1983; Pracha and Volk 2011) or among regions (Tzilivakis et al. 2005). They provide relevant knowledge for optimizing energy inputs in crop production. Energy returns on investment are also widely used to analyze the potentials of biofuels to replace fossil fuels, generally displaying the much lower EROI of biofuels in comparison to fossil fuels (e.g., Hammerschlag 2006).
3. A number of studies compare energy efficiency in conventional versus organic or other alternative farming practices (Refsgaard et al. 1998; Dalgaard et al. 2001; Gomiero et al. 2008; Atlason et al. 2015), including historical practices (Cussó et al. 2006). They generally find that the higher energetic output of conventional agriculture is achieved at a lower energy return on investment. These studies usually operate at the farm or crop level, and accounting procedures differ among studies; therefore, comparability and generalizability of results is limited.

These diverse approaches to energy efficiency in agricultural production offer relevant insights, but two major limitations prevail: firstly, methodological differences exist among most studies, inherent to the accounting of EROI indicators in general (Murphy et al. 2011), and agroecosystems energetics in particular (Giampietro et al. 1992). Differences owe to different research interests and resulting allocation procedures for energy inputs (Hall et al. 2011) and outputs. Therefore, the results of individual case studies remain largely context-specific. With a few important exceptions (Conforti and Giampietro 1997; Arizpe et al. 2011), systematic comparisons among energetic profiles in different cases are lacking. Secondly, studies on the energy efficiency of agricultural production focus almost exclusively on the ratio of final products to non-renewable energy inputs. While many include labor as energy input and some include feed imports, so far, local biomass inputs into agroecosystems, such as local feed or reploughed biomass, have been entirely neglected. Disregarding biomass reuse in agroecosystems impedes tracing long-term changes in agroecosystem energetics, starting in time periods when this was the major energy input to agroecosystems.

In this contribution, we close research gaps in both long-term socio-ecological research and research on agroecosystem energy analysis. We compare and discuss consistent long-term data of regional agroecosystem energy flows and energy returns on investment, including not only non-renewable but also organic energy inputs. We build on recent methodological advances to systematically account for different energy inputs in agroecosystems (Galán et al. 2016; Tello et al. 2016), which are suitable for long-term analysis. We use seven regional long-term case studies in Europe and North America to describe general features of what we call an energy transition in agroecosystems.

In the following section, we introduce the case studies, methods, and data sources used. In the “Results and discussion” section, we first describe the temporal trends in agroecosystem energy flows assessed and then portray general features of advanced organic and industrialized agroecosystems and the factors that differentiate the case studies. We conclude by proposing future lines of research.

## Case studies, methods and data

**Fig. 1:**
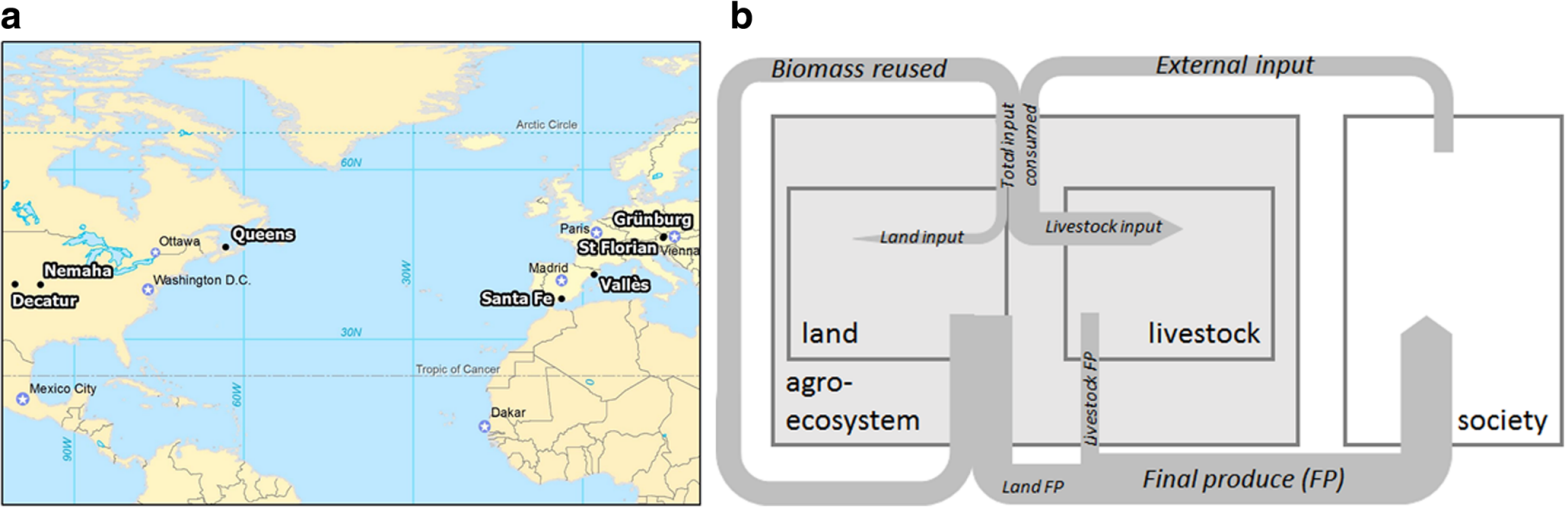
**(a)** Location of case studies and (**b)** conceptual framework of energy flows accounted

This study draws on consistent agroecosystem energy flow accounts from seven regional case studies on both sides of the Atlantic at three to five time points in the nineteenth and twentieth centuries, many of which are presented in this Special Issue (Cunfer et al. 2017; Gingrich et al. 2017; Marco et al. 2017; Guzmán and González de Molina 2015). The case studies, despite not being exhaustive, were selected to represent relevant environments in Europe and North America: Central European lowland and prealpine agriculture (St. Florian and Grünburg, Austria), Western Mediterranean agriculture focusing on vineyards (Vallés, Catalonia, Spain) and irrigated crops (Santa Fe, Andalusia, Spain), maritime frontier agriculture (Queens, Prince Edward Island (PEI), Canada), and grassland frontier agriculture (Nemaha and Decatur, Kansas, USA). Many other important landscapes and cultivation practices in Europe and North America are excluded from the analysis. Figure 1a presents the location of the case studies, and Table 1 provides general biogeographic features of the case studies.

**Table 1:** Biogeographic features of the case study regions. Climate data are derived from the respective nearest weather station and refer to current time periods: HISTALP database (St. Florian and Grünburg), Atles Climàtic de Catalunya (Valles), Climatedata.eu (Santa Fe), Usclimatedata.com (Decatur and Nemaha), and climate.weather.gc.ca (Queens). Potential NPP was roughly assessed based on climate data according to Lieth (1975)

Case study region		Province and country	Rainfall [mm/year]	Mean temp. [°C]	Potential NPP [g DM/m^2^/year]	Time period	No. of time points	Previous work on the region
Europe	St. Florian	Upper Austria, Austria	860	9.5	1276	1830–2000	5	Gingrich et al. (2017)
	Grünburg	Upper Austria, Austria	1000	8.9	1309	1830–2000	5	Gingrich et al. (2017)
	Vallés	Catalonia, Spain	700	13.9	1089	1860–1999	3	Galán et al. (2016); Tello et al. (2016); Marco et al. (2017)
	Santa Fe	Andalusia, Spain	358	14.7	618	1904–1997	3	Guzman and Gonzalez de Molina (2009); Guzmán and González de Molina (2015)
North America	Queens	Prince Edward Island, Canada	1158	5.7	1038	1880–1995	4	MacFadyen (2016); Supplementary Information section 1
	Nemaha	Kansas, USA	860	11.4	1276	1880–1997	4	Cunfer et al. (2017)
	Decatur	Kansas, USA	525	10.7	861	1880–1997	4	Cunfer et al. (2017)

The case studies vary greatly in terms of climatic conditions and agricultural structure, and display distinct trajectories over time. They also differ substantially regarding the degree of administrative integration (e.g., one county or several villages) as well as the area extent, the North American case studies exceeding the European ones by factors of up to 50. Still, each case study represents an agricultural landscape emerging from the specific local biogeographic conditions and the sum of agricultural and forestry practices of farmers operating there at a given point in time.

The European case studies in both Austria and Spain were characterized by mixed farming in the nineteenth century, with cropland covering more than 50% of farmland area, and livestock densities distinctly lower in Spain than in Austria (Table 2). By the end of the twentieth century, European agroecosystems specialized in cropping (Santa Fe, St. Florian), pig rearing (Vallés), and grassland-based cattle rearing (Grünburg). The North American cases show distinct differences from those in the Old World. For instance, nineteenth century population densities and farm laborers per unit of land were much lower than those in the European case studies. In Queens, PEI, cropland share and livestock density were comparable to European levels. At the time of the first data point in 1880, the two regions in Kansas had just commenced (Decatur) and finished (Nemaha) their pioneer periods, and both experienced cropland expansion during the late nineteenth century. By the end of the twentieth century, the distribution of land use types and livestock densities were at levels comparable to Europe, but population densities and numbers of farm workers per area remained well below the European levels.

**Table 2:** Agroecosystem features of the case study regions. Farmland area includes all land potentially used in agriculture or forestry, i.e., all land excluding unproductive and settlement areas. Data based on own calculations, see text

Case study region		Year	Population density	Farmers per km^2^ Farmland	Farmland	Cropland	Woodland	Grassland	Livestock density
			cap/km^2^	cap/km^2^	ha	% farmland	% farmland	% farmland	LSU500/km^2^ farmland
Europe	St. Florian, AT	1864	92.4	40	5008	65.1	17.9	17.1	54.5
		1950	172.5	23	8000	57.0	18.2	24.8	50.6
		2000	389.2	10	6733	76.6	18.1	5.2	32.4
	Grünburg, AT	1864	88.0	27	5924	38.9	27.4	33.7	39.8
		1950	78.0	22	10,065	30.7	22.4	46.8	49.1
		2000	84.5	15	8700	31.6	30.5	37.8	98.6
	Vallés, ES	1860	64.1	17	12,037	56.1	36.4	7.5	7.2
		1956	100.8	10	11,680	36.5	42.7	20.7	9.0
		1999	326.7	3	9323	23.4	72.9	3.7	241.1
	Santa Fe, ES	1904	187.3	44	3782	80.3	1.4	18.3	22.0
		1934	242.1	59	3601	84.1	6.2	9.7	38.0
		1997	320.9	17	3569	81.8	12.3	5.9	37.8
North America	Queens, CA	1880	24.3	11	170,193	48.4	35.0	16.6	26.3
		1950	21.6	6	166,811	41.5	37.0	21.5	30.3
		1995	36.5	0.8	164,527	43.4	47.9	8.8	37.3
	Nemaha, USA	1880	6.6	3	161,415	28.3	5.3	66.5	27.3
		1954	7.8	3	175,184	62.3	5.1	32.6	33.3
		1997	5.8	1	176,522	56.8	4.9	38.3	36.9
	Decatur, USA	1880	1.9	1	162,179	4.3	0.6	95.2	2.3
		1954	2.7	0.8	223,599	58.3	0.7	41.0	12.6
		1997	1.5	0.2	222,170	53.0	0.9	46.1	22.1

Three major energy flows through agroecosystems were accounted (Galán et al. 2016; Tello et al. 2016, Fig. 1b):

1. **Final produce** comprises all biomass products from the regional agroecosystem which are used by the local community or sold to markets, including crops and wood derived from the land (land final produce) and livestock products (livestock final produce).
2. **External inputs** encompass energy embodied in labor, household wastes, non-local biomass entering the agroecosystem (market feed or seeds), and industrial energy inputs (energy embodied and used in machinery, mineral fertilizers, pesticides, and electricity). External inputs are usually connected to economic costs and, depending on their composition, may result in a variety of environmental impacts, most prominently the emission of CO_2_ from fossil energy use, or the use of external land for biomass imports.
3. **Biomass reused** includes local reinvestments into the agroecosystems, such as livestock feed and litter, local seeds, and stubble burned or buried in soils (Guzmán and González de Molina 2015; Tello et al. 2016). Recycling biomass flows within the agroecosystem entails different environmental impacts than using external inputs and may, below a certain level, even contribute to ecosystem complexity (Marull et al. 2016). This energy flow is not considered in most studies accounting for agroecosystem energy efficiencies and offers new insights on the transformation of agroecosystems in the course of industrialization.

The accounted flows are used to generate three interrelated energy return on investment indicators, or EROIs (Galán et al. 2016): External final EROI (EFEROI) is the ratio of final produce to external inputs (Eq. 1). EFEROI considers most of the energy inputs that are accounted in traditional agricultural energy analyses (Dalgaard et al. 2001; Schramski et al. 2013; Atlason et al. 2015), but excludes local feed inputs or manure.

1 $$ \mathrm{External}\  \mathrm{final}\  \mathrm{EROI}\ \left(\mathrm{EFEROI}\right)=\frac{\mathrm{Final}\  \mathrm{produce}}{\mathrm{External}\  \mathrm{inputs}} $$

In order to investigate the important role of locally redirected energy flows, we define the internal final EROI (IFEROI) as the ratio of final produce to biomass reused (Eq. 2). IFEROI thus refers to the “efficiency with which intentionally recycled biomass is transformed into a product that is useful to society” (Guzmán et al. 2017).

2 $$ \mathrm{Internal}\  \mathrm{final}\  \mathrm{EROI}\ \left(\mathrm{IFEROI}\right)=\frac{\mathrm{Final}\  \mathrm{produce}}{\mathrm{Biomass}\  \mathrm{reused}} $$

The third indicator, final EROI (FEROI), is the ratio of final produce to total inputs consumed (i.e., the sum of external inputs and biomass reused) (Eq. 3).

3 $$ \mathrm{Final}\kern0.3em \mathrm{EROI}\kern0.3em \left(\mathrm{FEROI}\right)=\frac{\mathrm{Final}\kern0.3em \mathrm{produce}}{\mathrm{Total}\kern0.3em \mathrm{inputs}\kern0.3em \mathrm{consumed}}=\frac{\mathrm{Final}\kern0.3em \mathrm{produce}}{\mathrm{Biomass}\kern0.3em \mathrm{reused}+\mathrm{External}\kern0.3em \mathrm{inputs}} $$

Within the agroecosystem, we differentiate between energy entering the land system (seeds and residues inserted in soils, fuels, fertilizers, manure, pesticides, and labor for cultivation), and energy entering the livestock system (feed, litter, electricity, labor for livestock). Then, by dividing land final produce and livestock final produce by land total inputs and livestock total inputs, respectively, we obtain land EROI (Eq. 4) and livestock EROI (Eq. 5).

4 $$ \mathrm{Land}\  \mathrm{EROI}=\frac{\mathrm{Land}\  \mathrm{final}\  \mathrm{produce}}{\mathrm{Land}\  \mathrm{total}\  \mathrm{inputs}} $$

5 $$ \mathrm{Livestock}\  \mathrm{EROI}=\frac{\mathrm{Livestock}\  \mathrm{final}\  \mathrm{produce}}{\mathrm{Livestock}\  \mathrm{total}\  \mathrm{inputs}} $$

The database used in this paper builds on case studies which have been published in recent papers (Cunfer et al. 2017; Gingrich et al. 2017; Marco et al. 2017; Guzmán and González de Molina 2015). Data on Queens, PEI, Canada, have not yet been published. A brief regional description, and a source and method documentation of this case study are provided in the Supplementary Information’s section 1.

For all case studies, the most relevant data sources include region-specific agricultural censuses and cadastral records, detailing land use, population and livestock numbers, yields, agricultural labor force, and agricultural machinery. National or regional information was used and downscaled to the respective regions in order to fill data gaps (e.g., on pesticides, fertilizer, or electricity use). Data from census statistics were available either as archival material, as individual publications, or as databases from the respective national statistic agencies. Flows not reported in statistical records were estimated. The most important such flows are straw, which was assessed based on grain harvest and harvest indices if not reported in statistics, and grazed biomass, which was estimated based on feed supply and feed demand by local livestock, as well as information on pasture land and pasturing practices.

Energy flows were assessed by converting flows of biomass into their energy content (Guzman et al. 2014) and by calculating the embodied energy in external inputs, accounting for historical changes in industrial efficiency (Aguilera et al. 2015). For the European cases, labor was accounted as the energy content of gross food intake per unit of time worked managing the agroecosystem, and embodied energy in food processing, transport, and cooling was considered in the twentieth century. For the North American cases, labor was estimated as 2 GJ/person/year for each working age laborer (Cunfer and Krausmann 2015). For reasons of cross-case study consistency, minor differences exist between the previously published data and the ones presented here, in particular in the Kansas case studies where per-area data refer to “farmland” here, i.e., land used for agricultural or forestry production (and to total land area in Cunfer et al. 2017), and biomass reused includes stubble plowed into soils here (and excludes it in Cunfer et al. 2017).

For this comparative analysis, the agroecosystem energy flow data were analyzed with regard to potential explanatory variables, such as population density, livestock density, land use distribution, or the composition of different energy flows. Despite not being large enough for statistical analyses, the consistent data set of seven very diverse case studies enabled us to develop general hypotheses on land use intensification which go beyond the individual cases.

While complying with a consistent methodology, some caveats need to be considered given variations among the case studies. First, the choice of case studies is not representative for global trends. We thus use the sample to propose some general features of industrializing agriculture in Europe and North America; some of which may hold true also for other world regions. Secondly, due to the difference in case study areas, scale-dependent indicators need to be interpreted carefully. A smaller share of biomass reused in a smaller case study may be the result of the chosen system boundary, rather than actually lower regional energy transfers. The inverse holds true for external inputs. We compare only per-area values to level off differences in area extent, but we reckognize the potential bias caused by different case study sizes.

## Results and discussion

### Trajectories of the agroecosystem energy transition

**Fig. 2:**
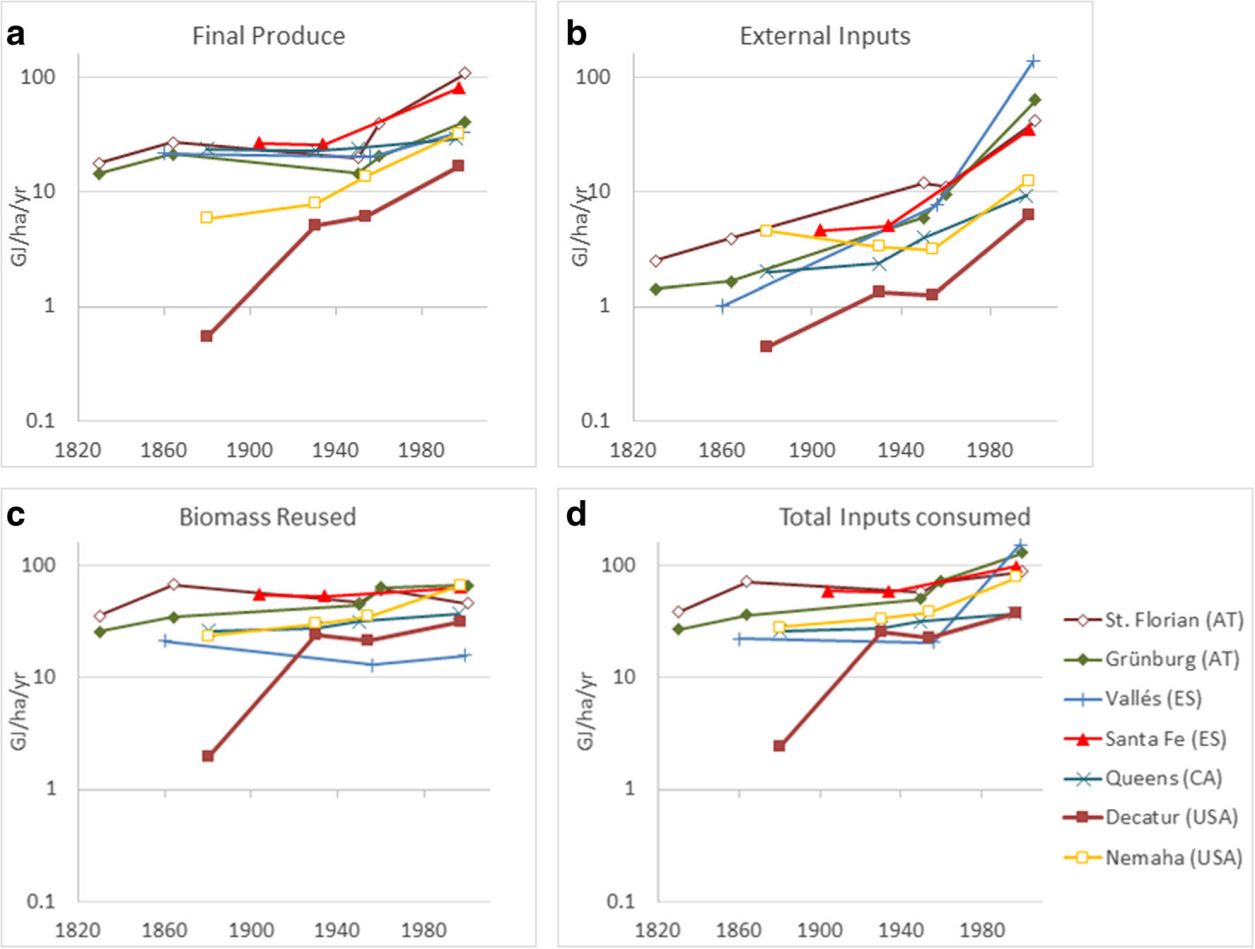
Agroecosystem energy outputs (final produce) (**a**) and inputs (external inputs (**b**), biomass reused (**c**), and total inputs consumed (**d**), i.e., the sum of external inputs and biomass reused); log scale

Both final produce and external inputs increased in all case studies over the time period investigated: Final produce grew in all case studies between the first and last data points, sometimes interrupted by lower productivity in intermediate time points (Fig. 2a). The smallest increase to final produce between the first and last data points was 24% in Queens, Canada, where forest products, which saw little productivity change, contributed over 90% of final produce in the nineteenth century and still 53% in 1995. The strongest increase in final produce was in Decatur (factor 31), USA, where the first data point reflects early pioneer conditions during the first years of European settlement. The combination of very low cropland extent and low wood extraction due to ecological conditions (natural grasslands) explain the low value in 1880 (Cunfer et al. 2017). At all time points, the lowest levels of final produce were in the Kansas case studies. In the twentieth century, the highest final produce (around 100 GJ/ha/year) was achieved in Santa Fe, Spain, and St. Florian, Austria, where non-edible biomass production (wood and straw) contributed significantly to final produce (Gingrich et al. 2017).

External inputs of energy increased even more strongly than final produce in all case studies, following almost exponential trajectories (Fig. 2b). The most pronounced increase occurred in Vallés (factor 136), while the smallest increase took place in Decatur (factor 16). After the mid-twentieth century, external inputs reached levels comparable to final produce. Biomass reused (Fig. 2c) increased or remained stable in all the case studies in the time period investigated, with the exception of Vallés, where it declined by 26% due to the abandonment of traditional biomass intensive fertilizing techniques. The flows of biomass reused were the most important energy input into agroecosystems throughout the time period in most case studies and retained levels comparable to those of external inputs at the end of the twentieth century. Trends of total inputs consumed in agroecosystems (Fig. 2d) therefore resemble those of biomass reused until the mid-twentieth century. Only towards the end of the twentieth century did total inputs consumed increase due to external inputs, while biomass reused remained stable. The methodology adopted here displays that the agroecosystem energy transition is characterized by a shift from largely local energy inputs (biomass reused dominates total inputs consumed) to a combination of similar amounts of local and external energy inputs (biomass reused and external inputs are similar). From a socio-ecological perspective, the increased inputs from modern energy carriers added to local agroecosystem biomass reuses, rather than replacing them.

**Fig. 3:**
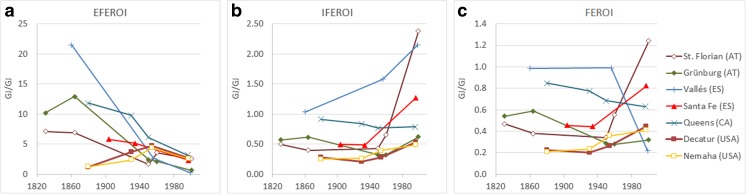
Energy returns on investment (EROIs): (**a)** external final EROI (EFEROI, ratio of final produce to external inputs), (**b)** internal final EROI (IFEROI, ratio of final produce to biomass reused), **c** final EROI (FEROI, ratio of final produce to total inputs consumed, i.e., the sum of external inputs plus biomass reused)

The changes in energy inputs and outputs entailed specific trajectories of EROIs (Fig. 3). EFEROI (final produce to external inputs) declined in almost all case studies, quite in line with previous findings on agriculture’s growing demand of external energy inputs (Pimentel et al. 1973; Smil 2000). In the nineteenth century, EFEROI values ranged between 7 and 12 in many case studies, i.e., final produce exceeded external inputs by these factors. EFEROI was distinctly lower in Decatur and Nemaha, USA (slightly above 1), and higher in Vallés (around 22). In most case studies, the strong decline of EFEROI in the late nineteenth and early twentieth centuries reflects increasing external energy inputs while final produce did not grow much.

After World War II, EFEROI converged to values between c. 2 (St. Florian) and 6 (Queens), and by 2000, it declined in all case studies, to between 0.2 (Vallés) and 3.2 (Queens). EFEROI was below 1 in two case studies in 2000 (Vallés and Grünburg), indicating that farmers were investing more energy through external inputs than they got back as final produce. In all other case studies, growth in final produce kept pace with growing external inputs in the second half of the twentieth century. Our EFEROI values for the USA and Canadian case studies are similar to the ones obtained at the national scale (Steinhart and Steinhart 1974; Hamilton et al. 2013), though the empirical basis differs in scale and scope.

IFEROI, i.e., final produce per unit of biomass reused (Fig. 3b), was lower than EFEROI in all case studies until the mid-twentieth century, given that biomass reused exceeded external inputs. Towards the late twentieth century, however, IFEROI increased in most case studies, because final produce grew strongly while biomass reused remained relatively stable. The only exception is Queens, Canada, where IFEROI declined in the late twentieth century. The case studies in which IFEROI increased above 1 focused on intensive cropping (St. Florian), intensive pig rearing based on non-local market feed (Vallés), or wood plantations (Santa Fe). In all other regions, IFEROI remained below 1, i.e., biomass reused was larger than final produce even in industrialized agriculture. This highlights the fact that local biomass continues to be an important energy input in industrialized agroecosystems, which has not been replaced, but merely complemented, by industrial, fossil fuel-based energy inputs.

FEROI, i.e., final produce per unit of total inputs consumed (external inputs plus biomass reused), shows no clear temporal trend across case studies (Fig. 3c). With one exception (Decatur), FEROI decreased or remained stable in the period before the mid-twentieth century. Around this time, mechanization of agriculture already required more energy inputs, but land productivity had not increased on a large scale. This suggests farmers adopted tractors to save labor, not to increase production. In the second half of the twentieth century, FEROI developed differently in the case study regions, declining slightly or strongly in some, while increasing more or less strongly in others. The most extreme cases of change after World War II were in Vallés, where specialization on import-dependent pork production caused FEROI to decline strongly, and St. Florian, where FEROI increased strongly due to specialization on high-yielding crop production and a redirection of straw from local reuse to external markets. The fact that the most extreme EROI values were reached in the European case studies may be in part linked to the fact that the regions investigated in Europe were smaller than the North American ones and that specialization processes possibly occurring in North America are evened out through the larger area investigated.

The overall inconclusive trend of FEROI demonstrates two important features of the agroecosystem energy transition: (1) Increased productivity came as a result of more external energy inputs, while retaining some internal biomass reuses. (2) Particularly in the second half of the twentieth century, regional agroecosystems changed in very diverse ways, resulting from increased regional specialization. Energy flow and EROI numbers for all case studies are provided in the Supplementary Information, section 2, and trajectories of energy input and output intensities of land use are discussed in the Supplementary Information, section 3.

### Comparing European advanced organic agriculture with the North American frontier in the late nineteenth century

We now compare the case studies in the late nineteenth and early twentieth centuries (i.e., 1860 in Vallés, 1864 in the St. Florian and Grünburg, and 1904 in Santa Fe) with the 1880 North American case studies. Despite the 40-year time difference between the first and last data points, the European case studies reveal characteristics of advanced organic agriculture, while the North American cases are characterized by frontier conditions.

Drawing from Wrigley’s notion of an “advanced organic economy” (Wrigley 1990), we define advanced organic agriculture as farming practices that (1) used very little fossil fuel, (2) relied on local resources mainly, and (3) raised land productivity through increasing labor inputs. Despite remaining in the biomass energy regime, advanced organic economies began to participate in modern, supra-regional market exchange. According to scholars like Boserup (1965, 1981) and Geertz (1963), close links existed between population density and land use intensity under such conditions. The frontier conditions in North America were somewhat different, because already in 1880, they displayed both fossil energy input and more significant market integration. We define late nineteenth century North American frontier agriculture as abundant, recently colonized land with a limited agricultural labor force.

We investigate the nineteenth century data with the hypothesis that with increasing population density, land productivity is increased at the expense of decreasing labor productivity and decreasing IFEROIs. If so, the increased inputs of labor and biomass reused would outpace the resulting yield increases.

**Fig. 4:**
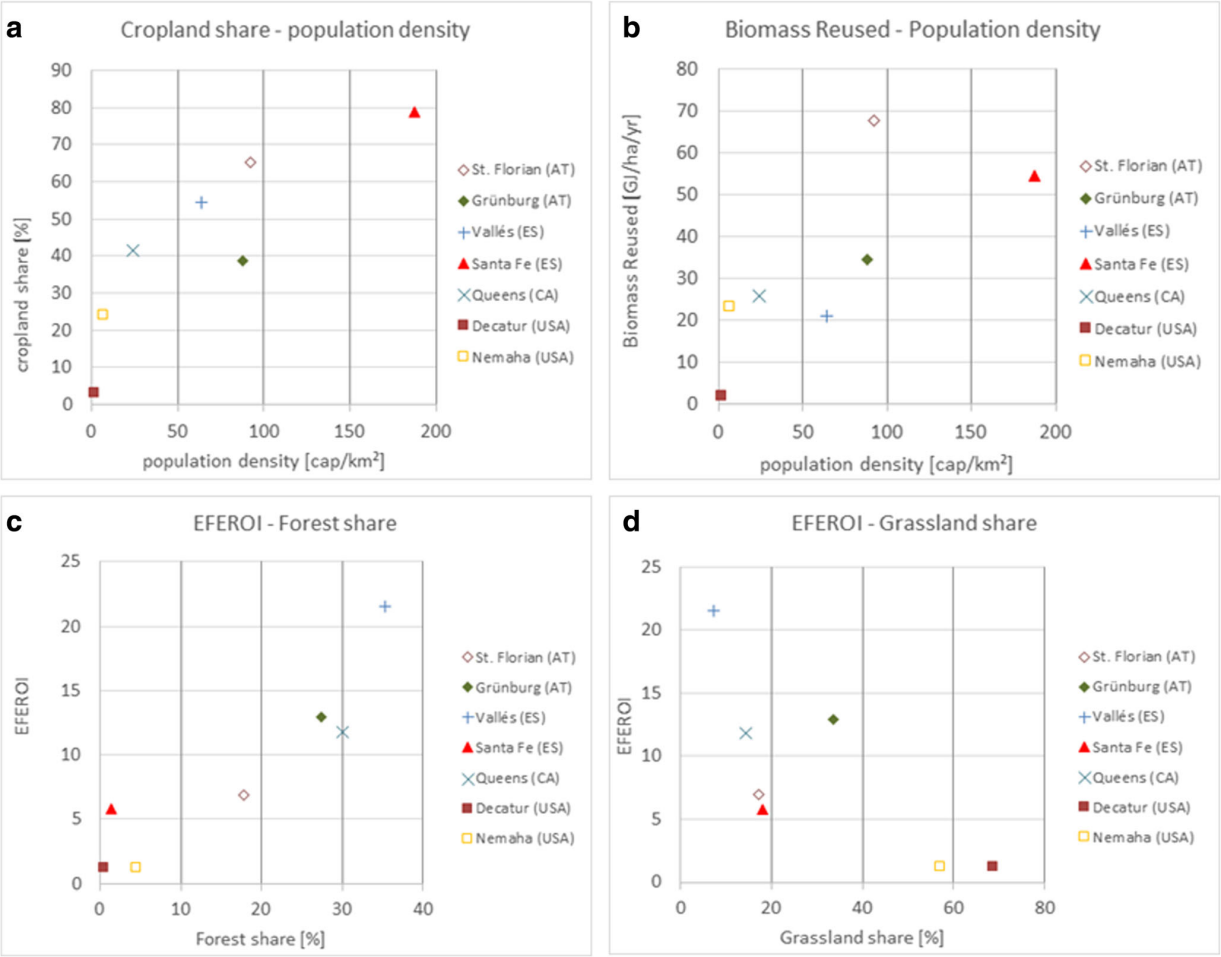
Features of advanced organic and frontier agroecosystems: the share of cropland in farmland area (**a**) and the amount of biomass reused (**b**) correlated positively with population density. However, energy efficiency (EFEROI) was more determined by other land use types, i.e., forest share (**c**) and grassland share (**d**)

Our case studies reveal a distinction between European advanced organic agroecosystems and those in North American frontier settings. For example, the lower population density in the North American case studies (2–24 cap/km^2^ as opposed to 64–187 cap/km^2^ in Europe; Table 2) coincided with a lower cropland share in North America (Fig. 4a). Only Grünburg stands out, where high population density at lower cropland shares resulted from regional manufacturing activities increasing population density.

The difference in cropland shares entailed different agroecosystem energy flows in European and North American agroecosystems. Final produce per area was higher in the European case studies (21–27 GJ/ha/year as opposed to 0.5 to 24 GJ/ha/year in North America), and so were energy inputs per unit of area, in terms of labor and biomass reused. Only external inputs were comparable in the North American regions, despite the larger size of North American case studies. The indicator biomass reused proves to be a good proxy for land use intensity in nineteenth century agroecosystems, as it is higher in those case studies of higher population density, higher cropland shares (Fig. 4b), and higher fertilization requirements. Where cropland extent was higher, more biomass was recycled either to livestock, generating manure, or directly into soils as in Vallés. St. Florian stands out here as a region of particularly high biomass reused, caused both by high straw availability and intensive livestock management (livestock kept in stables all year required more litter than livestock grazing at least parts of the year).

While the level of different energy flows correlated with population density, energy returns on investment show no clear interrelation with population density. Instead, energy returns on investment had to do with the land uses other than cropland. High forest shares were related to higher EFEROI values, and high pasture shares to low EFEROI values (Fig. 4c, d). The land use types other than cropland were greatly influenced by biogeographical factors such as climate and native vegetation. Extensive land use requiring little labor per unit area (that is, land use of low “input-intensity”; Erb et al. 2013) allowed for wood extraction in Queens and for rangeland livestock rearing in Kansas. Forests yield high energetic output at low input, while livestock rearing yields low final produce per unit of energy input. Therefore, in our accounting metrics, the lower energetic efficiency of regions with low forest shares (in our case studies: particularly in Kansas) was the result of low rainfall favoring specific management practices.

At a higher level of abstraction, the data allows for a thought experiment discerning two different types of land use intensification. In traditional European agriculture, where croplands were already covering high shares of suitable land, increases to agricultural production could only be achieved by intensification, which required more inputs of labor and biomass reused when no substantial external inputs were available, and resulted in stable or declining EROIs. This supports our initial hypothesis. In a frontier situation such as the one in Kansas, however, we see a different trajectory: cropland expansion at the expense of extensive livestock rearing may have yielded higher final produce at declining inputs of labor and biomass reused per unit of output, thus resulting in increasing energy returns on investment.

### Industrialized agroecosystems: high input, high output, and regional specialization

By the late twentieth century, some of the biophysical constraints to organic agriculture had lost their relevance. External energy sources had become available in substantial amounts, adding to local biomass flows. Due to low energy prices, energy turned from a biophysical constraint into just one of many economic factors influencing farmers’ farm management decisions. Our data suggest that agroecosystem energy efficiency in the late twentieth century depended greatly on the particular specialization path followed by agricultural production in each region.

**Fig. 5:**
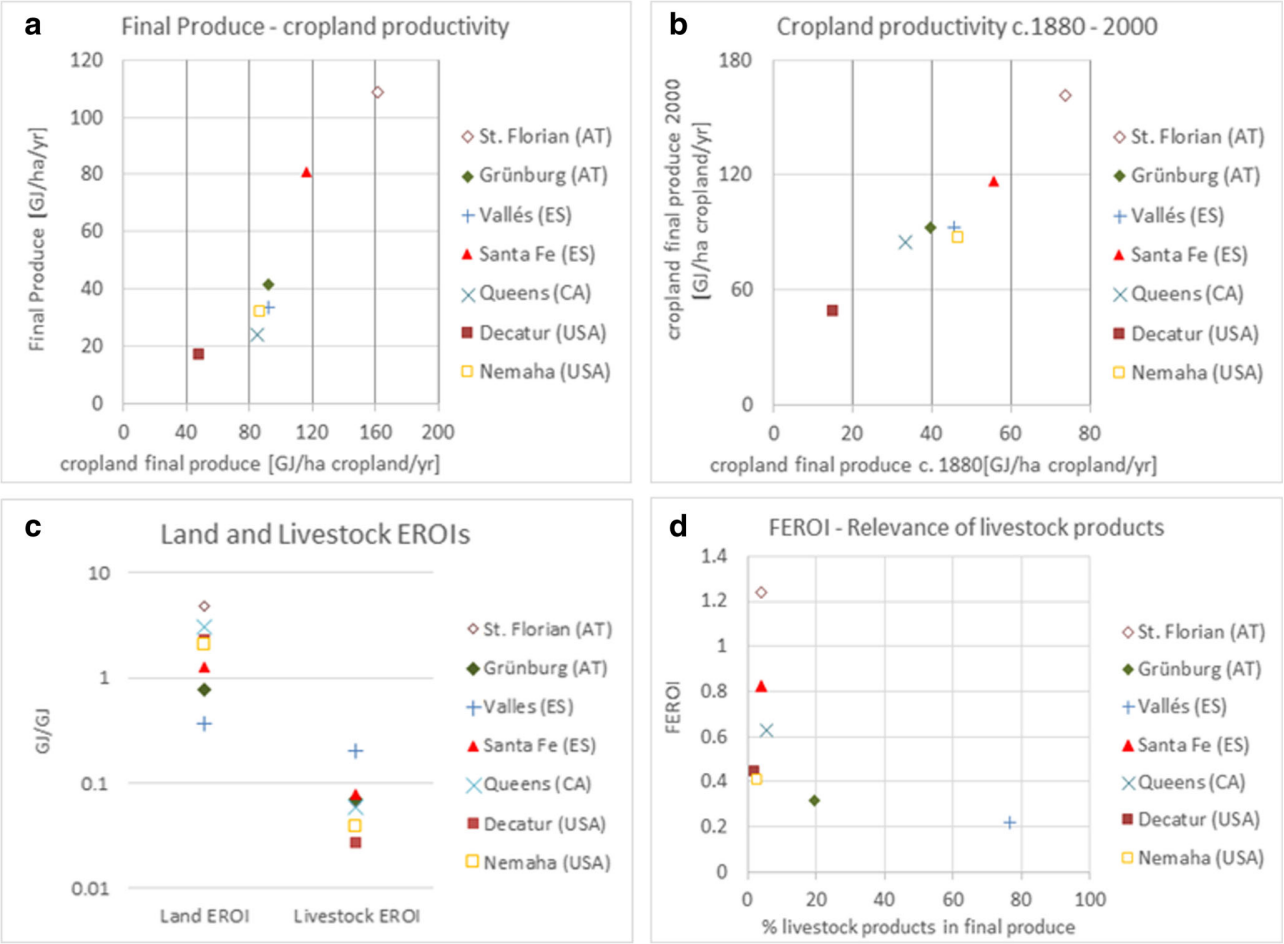
Features of industrialized agroecosystems: cropland productivity explains much of the final productivity (**a**) and is strongly linked to cropland productivity in advanced organic/frontier agriculture of the respective case study (**b**). (**c)** Energy efficiency differs greatly between land-based and livestock-based biomass production, log scale; (**d)** relevance of livestock products explains much of final EROI (FEROI)

The amount of final produce generated by industrialized agroecosystems was strongly related to cropland productivity in our case studies. Places where cropland final produce per cropland area was highest (St. Florian and Santa Fe) also exhibited the highest total final produce per total land area. Likewise, the regions with the lowest cropland productivity displayed the lowest overall land productivity (Fig. 5a). This reflects the fact that croplands contributed more than 80% to final produce in some case studies (St. Florian and the Kansas case studies), while livestock production or forestry compensated for the difference in the others. Cropland intensification took place particularly in those regions with favorable biogeographic conditions (or, as in Santa Fe, with conditions suitable for irrigation). Figure 5b illustrates that cropland productivity in 2000 correlated strongly with cropland productivity in the late nineteenth century. In most case studies, cropland productivity increased by a factor 2 to 3 between the late nineteenth century and the end of the twentieth century.

The level of energy inputs into each agroecosystem was related to a number of variables. In case studies with low livestock densities and intensive cropping (Santa Fe, Spain, and Decatur and Nemaha, USA), fuels for tractors and fertilizers dominated energy inputs. In the other case studies, external feed inputs for livestock were the most important energy inputs. Regions where livestock density was highest and livestock products contributed 20% or more to final produce (Grünburg and Vallés) featured the highest total inputs consumed (130 and 153 GJ/ha/year, respectively). Still, the two livestock regions display significant differences. In Grünburg, where steep topography obstructed industrialized cropping, grassland-based cattle rearing dominated. Important shares of feed were provided locally, and manure was returned to the land. Industrialized pig and poultry production in Vallés occurred more independently from local biogeographic conditions and was favored for socio-economic reasons. In the feedlots of Vallés, biomass reused played only a minor role, and external feed inputs exceeded livestock biomass reused by a factor 7 in 1999. The two cases demonstrate structural differences in energetic profiles depending on the type of livestock management, which have been displayed to be considerable, e.g., at the national scale in the USA (Pelletier et al. 2011).

While the levels of final produce were linked to cropland productivity, livestock played a bigger role in shaping energy inputs. Land-based biomass production (both on agricultural land and forests) and livestock production differ not only in terms of energy outputs and inputs but also in the ratio between the two, i.e., their energetic conversion efficiency. Figure 5c presents the indicators “Land EROI” and “Livestock EROI” in all our case studies in 2000. The comparison shows that energy conversion efficiency in land-based biomass production exceeded energy efficiency in livestock production by around a factor 10. The differences between the case studies can be attributed in part to differences in the composition of land use and livestock species. Livestock EROI was lowest in Decatur, USA, where cattle, the least energy-efficient major livestock species in our case studies, accounted for over 95% of standardized livestock units. In Vallés, where livestock rearing was dominated by pork production, and ruminants account for less than 10% of total livestock, livestock EROI was the highest. Similarly, land use composition explains part of the differences in land EROIs, with the highest value in St. Florian, where 77% of farmland was used as cropland, the most productive land use type in industrialized agroecosystems, and the lowest in Vallés (18% cropland).

The share of livestock products in final produce was an important factor shaping differences in FEROI (the ratio between final produce and total inputs consumed), as shown in Fig. 5d. With the high energy input requirements of livestock production and the relatively inefficient conversion to final produce, the relevance of livestock products significantly affected FEROI values. The potential for high energetic dependence on external inputs, and for providing remote markets (rather than local subsistence), allowed for strong and diverging specialization in industrialized agroecosystems. An agroecosystem like the one in Vallés, where feed demand greatly exceeded local feed production, would not have been possible under advanced organic conditions. Our data suggest that fossil fuel-based energy inputs loosened the links between population density and land use intensity. Instead, a combination of biogeographic conditions and socio-economic factors led to specialization of agroecosystems on particular production types, determining the energetic profiles of regional agroecosystems.

## Conclusions and outlook

In the course of industrialization, biomass production was increased at the expense of increasing amounts of modern energy inputs. Our analysis, based on the consistent comparison of seven regional case studies across the Atlantic, confirms this general observation and adds two major insights on what we call an agroecosystem energy transition. (1) Energetic transfers within agroecosystems (e.g., local feed and litter provision), which accounted for the largest fraction of agroecosystem energy inputs in advanced organic and frontier agriculture, remained a significant energy input throughout the period. This means that, despite growing external energy inputs, both industrial and biotic, overall energy efficiency of agroecosystems did not decline as much as suggested by previous work. (2) The factors explaining differences among agroecosystem energy efficiencies changed in the course of industrialization: in pre-industrial and frontier agroecosystems, these factors were mostly biophysical (population density, suitability for wood extraction versus grazing). In industrialized agroecosystems, however, regional specialization on specific agricultural production processes, partly favored by biogeographic conditions but partly by socio-economic factors, determined energetic profiles of regional agroecosystems.

Based on our findings, we identify two important lines of future research:

1. The results here are based on a sample of agroecosystems representative of specific agroecological and geographical zones with a specific land use history. Future research investigating other regions with different agricultural practices and different land use histories, and more samples, e.g., including Asian or African case studies, and plantation, rice cultivation or rangeland systems, are required to shed light on agroecosystem energy trajectories under different agroecological and socio-cultural conditions. In addition, work at different scales, ranging from the farm household to the village, the province, country, or world region, will allow for identifying the effects of scale-specific trajectories, such as regional specialization.
2. Exploring the links of agroecosystem energy transitions to both socio-economic and ecological processes promises to yield important insights. At the regional scale, landscape metrics, soil nutrient balances, greenhouse gas emission balances, or the human appropriation of net primary production may help explain different types of ecological pressures exerted by different energy profiles. Agroecological EROI indicators (Guzmán et al. 2017; Guzmán and González de Molina 2015) provide insights about energy flows related to the state of fund elements of agroecosystems and thus reveal details of their ecological sustainability. Incorporating a socio-economic perspective on the other hand, e.g., by investigating prices of agricultural products or production factors, or analyzing the role of political decision-making related to land use change, informs about the complex rationale of farmers’ decision-making in particular during agricultural specialization of the twentieth century. Ultimately, such research could offer insights on sustainable land use intensification by identifying energy-efficient agroecological optimization strategies which meet social needs while sustaining agroecological functioning.

## Electronic supplementary material

ESM 1 (PDF 553 kb)
