# Supplementary material for: Agroecosystem energy transitions in the old and new worlds: trajectories and determinants at the regional scale
Source: Reg Environ Change. 2017 Dec 7;18(4):1089–101. doi: 10.1007/s10113-017-1261-y (PMC6560785; doi:10.1007/s10113-017-1261-y)
Supplement: Supplementary file 1 — (PDF 553 kb) [file 10113_2017_1261_MOESM1_ESM.pdf]

Supplementary Information

Agroecosystem energy transitions in the old and new worlds: trajectories and determinants at the regional scale

Simone Gingrich, Inés Marco, Eduardo Aguilera, Roc Padró, Claudio Cattaneo, Geoff Cunfer, Gloria Isabel Guzmán Casado, Joshua MacFadyen, Andrew Watson

1. Detailed description Prince Edward Island, Canada

Joshua MacFadyen

1.1. The region

This note outlines research on energy in agro-ecosystems in the Canadian province of Prince Edward Island (PEI) from 1880-1995. PEI is the smallest province in Canada, with only 143,000 residents, one small city, and 5,660 square kilometers of land in the southern Gulf of Saint Lawrence. The island's level terrain, well-drained soil, and stable climate made it more suitable for agriculture than most parts of Atlantic Canada. Farming and fishing have dominated PEI's economy since French Acadian settlement in the eighteenth century and intensive British settlement in the nineteenth and twentieth centuries. The island's dense mixed forest was one of the practical barriers to farm settlement, but used in conjunction with marsh hay and other estuarine resources, the forest provided shelter, feed, and materials necessary for a system of mixed animal husbandry in the organic regime (Hatvany 2001; Bittermann and McCallum 2016). In the late twentieth century, PEI farmers maintained this system of livestock, but they slowly began to replace beef and dairy with hog production. The recent period of industrial agriculture is known for a new focus on intensive crops (Arsenault 2016), but in energetic terms, a more significant transition occurred when farmers began to reduce their own internal cycling of feed from crops and pasture. Building on recent work in PEI's agricultural history (MacFadyen and Glen 2014; MacFadyen 2016a), this case study examines energy flows in the regional agroecosystem in the period from 1880 to 1995.

The county selected for this comparative research was Queens County, PEI. Queens was the most populated and most agriculturally productive of PEI's three administrative regions. It was also the home to the provincial capital, Charlottetown, although this city was removed from the energy analysis. The recent research covers the longer period of British settlement and recent modernization, but this study focuses on data gathered from four main time points (1880, 1930, 1950, and 1995) to trace major changes in agroecosystem energetics from early European settlement to recent industrialization and intensification processes.

1.2. Sources and methods

The Canadian *Census of Agriculture* is the primary resource for this study. These censuses were conducted at the same time as the regular census of population (decennially starting in 1881 and quinquennially starting in 1916). The Canadian census of agriculture provides information on a broad range of scales. Agricultural data were reported from national and provincial down to county (Census Division) and township (Census Subdivision) levels. The census bureaus also changed their reporting practices over time, and toward the end of the study period, data from other agencies like Statistics Canada are required to fill in some key energy flows.

The size and composition of the agroecosystem, including agricultural workforce and population, was determined through the land use data in the *Census of Agriculture*, where information on land used by farms was reported (Department of Agriculture 1883b; Canada Census Office 1934; Dominion Bureau of Statistics 1953; Statistics Canada 1997, CANSIM database). In addition, land use inventories created by the PEI departments of Forestry and Agriculture were used to trace non-farmed (in particular forested) land (Prince Edward Island Canada 2003a, b; MacFadyen and Glen 2014). Livestock units were determined from the quantity of each animal reported in the censuses and standardized by the historical (Gentilcore et al. 1993) and modern (FAO 2003) weights of each species.

The two primary categories of agro-ecosystem energy *outputs* are **Land Produce** (LP), i.e. all plant biomass consumed by the local farmers, sold to markets, or reinvested in the agroecosystem as Biomass Reused (see below), and **Livestock Barnyard Produce** (LBP), i.e. all final produce from livestock (for definitions of these terms, see e.g. Tello et al.(2016)). In Queens County LP from cropland was a relatively straightforward conversion of crop data from the censuses (Department of Agriculture 1883a; Canada Census Office 1934; Dominion Bureau of Statistics 1953; Statistics Canada 1997, CANSIM database), and converted based on historical assumptions available in Guzman et al. (2014).

Woodland produce data was developed using Census of Agriculture reports on products of the forest (especially firewood and fencing/lumber used on the farm, see Department of Agriculture 1883; Canada Census Office 1934; Dominion Bureau of Statistics 1953). We then refined those firewood data according to new estimates in biomass energy history (MacFadyen 2016b). In 1995, the woodland production was determined by provincial forestry and firewood consumption surveys (Smith Green & Associates 1995; Prince Edward Island Canada 2003c). Pastureland produce was determined by a combination of census data on pasture availability and estimates of regional productivity (Department of Agriculture 1938), and results were checked against ruminant consumption based on feed balances from the calculations of Biomass Reused (see below).

LBP is a measure of all the energy contained in livestock products, including butter, milk, and eggs, and in slaughtered animals calculated by the slaughter weight of each species. The Canadian census data allow an accurate measurement of most animal products, and Guzman et al. (2014) provide conversion factors to assess energy flows.

For the calculation of **Biomass Reused** (BR), we used estimates of the demand for seed, feed, and litter that were based on the available census data on crop and livestock production. We calculated this demand using conversion factors that are informed by historical analysis of the local and regional variations on feeding livestock. After the demands were calculated, they were compared with available litter, forage and feedstuffs to determine if local farms produced enough to account for the demands internally. Any demands that exceeded local production, and even some that did not, were considered as external inputs.

In order to determine cropland BR, the amount of seed needed for sowing the following season's crop was deduced from Final Produce. For roots that meant 10 percent, but for grains it ranged from 5 to 15 percent, depending on the crop. Determining the Livestock-Barnyard BR is one of the most complex processes of the energy analysis. It involves many historical assumptions about the animals present in an agroecosystem and the sources and varieties of the feed they consumed in the time point under study. In Queens County, it was particularly difficult to understand BR, because of the nineteenth century food and feed shortages outlined in earlier work (MacFadyen 2016a). The solution involved a complex feed

balance that accounted for all of the possible feed types in the county and adjusted for period-specific feeding rates (and animal survival rates) based on historical research. For instance, a significant reduction of pasture land in the late twentieth century meant there would have been a shortage of grazed biomass in 1995. However, increased hay (and some feed grain) production accounted for the difference. Historical estimates of the quantity and apportionment of each feed type were derived from a variety of sources (Armsby and Moulton 1925; Jennings 1953; Statistics Canada 1982; Lucci et al. 2013 CANSIM database).

For **External Inputs** (EI) the most important variable to know in the early periods in Queens County was human labor which was estimated based on the Census of Population (Department of Agriculture 1883a, 1884). Beginning in 1930, additional information is available for hired farm workers (Canada Census Office 1934). Later in the twentieth century, other external inputs become increasingly important, particularly the energy intensive forms such as artificial fertilizers, machinery, and fossil fuels (Dominion Bureau of Statistics 1953; Statistics Canada 1982). These were converted to GJ/ha based on their embodied energy as specified in Aguilera et al. (2015) and other sources (Audsley et al. 1997; Dhungana 2007). The 1995 county-level fertilizer consumption was estimated by the Statistics Canada data on the N, P, & K fertilizers imported for agriculture in 1995/1996 divided by Queens County's share (16%) of the region's total area treated by commercial fertilizers (P.E.I. Statistics Bureau 2013, CANSIM database). Fossil fuel and electricity consumption were determined by the relative amount of expenditure on these inputs by Queens County farmers as reported in the 1995 census.

1.3. References

Aguilera E, Guzmán G, Infante-Amate J, Soto D, Garcia-Ruiz R, Herrera A, Villa I, Torremocha E, Carranza G, Gonzalez de Molina M (2015) Embodied energy in agricultural inputs. Incorporating a historical perspective. Documentos de Trabajo 1507, Sociedad Española de Historia Agraria (SEHA)

Armsby HP, Moulton CR (1925) The animal as a converter of matter and energy; a study of the role of live stock in food production. Book Department, The Chemical Catalog Company, inc., New York

Arsenault J-P (2016) Agriculture and the Environment on Prince Edward Island, 1969-2014: An Uneasy Relationship. In: MacDonald E, MacFadyen J, Novaczek I (eds) Time and a Place: An Environmental History of Prince Edward Island. McGill-Queens University Press, Montreal, pp 195–217

Audsley E, Alber S, Clift R, Cowell S, Crettaz P, Gaillard G, Hausheer J, Jolliet O, Kleijn R, Mortensen B, Pearce D (1997) Harmonisation Of Environmental Life Cycle Assessment For Agriculture. European Comm., DG VI Agriculture

Bittermann R, McCallum M (2016) “One of the Finest Grass Countries I Have Met With”: Prince Edward Island’s Colonial-Era Cattle Trade. Agricult Hist 90:173. doi: 10.3098/ah.2016.090.2.173

Canada Census Office (1934) Seventh Census of Canada, 1931, Prince Edward Island Census of Agriculture. J. Patenaude, Ottawa

Department of Agriculture (1883a) Census of Canada, 1880-81, Recensement du Canada, 1880-81 Vol 1. MacLean, Roger & Co, Ottawa

Department of Agriculture (1883b) Census of Canada, 1880-81, Recensement du Canada, 1880-81 Vol 3. MacLean, Roger & Co, Ottawa

Department of Agriculture (1884) Census of Canada, 1880-81, Recensement du Canada, 1880-81 Vol 2. MacLean, Roger & Co, Ottawa

Department of Agriculture C (1938) Pasture Improvement in Eastern Canada. Government Printer, Ottawa

Dhungana BR (2007) Economic modeling of bioenergy crop production and carbon emission reduction in Illinois. University of Illinois at Urbana-Champaign

Dominion Bureau of Statistics C (1953) Ninth Census of Canada, 1951, Neuvieme recensement du Canada, Vol 6: Agriculture. Government Printing Office, Ottawa

FAO (2003) Compendium of Agricultural-Environmental Indicators, Annexe 2: Definitions. Food and Agriculture Organization of the United Nations

Gentilcore RL, Measner D, Walder RH (eds) (1993) Historical Atlas of Canada: Volume II: The Land Transformed, 1800-1891. University of Toronto Press, Toronto

Guzman G, Aguilera E, Soto D, lnfante-Amate J, Garcia-Ruiz R, Herrera A, Villa I, Gonzalez de Molina M (2014) Methodology and conversion factors to estimate the net primary productivity of historical and contemporary agroecosystems. Documentos de Trabajo 1407, Sociedad Española de Historia Agraria (SEHA)

Hatvany MG (2001) “Wedded to the Marshes”: Salt Marshes and Socio-Economic Differentiation in Early Prince Edward Island. Acadiensis 30:40–55.

Jennings RD (1953) Animal units of livestock fed annually, 1909-10 to 1952-53. US Department of Agriculture, Agricultural Research Service, Washington

Lucci G, Carlson B, Shepherd M (2013) Variability of Dry Matter Percentages of Brassica Forages - Getting Feed Estimates Right. Fertilizer & Lime Research Centre, Massey University, Palmerston North

MacFadyen J (2016a) The Fertile Crescent: Agricultural Land Use on Prince Edward Island, 1861-1971. In: MacDonald E, MacFadyen J, Novaczek I (eds) Time and a Place: An Environmental History of Prince Edward Island. McGill-Queen’s University Press, Montreal, pp 161-194

MacFadyen J (2016b) Hewers of Wood: A History of Wood Energy in Canada. In: Sandwell RW (ed) Powering Up Canada: A History of Power, Fuel, and Energy from 1600. McGill-Queen’s University Press, Montreal, pp 129–161

MacFadyen J, Glen W (2014) Top-down History: Delimiting Forests, Farms, and the Census of Agriculture on Prince Edward Island Using Aerial Photography, ca.1900-2000. In: Bonnell J, Fortin M (eds) Historical Geographic Information Systems in Canada. University of Calgary Press, Calgary, pp 197–223

P.E.I. Statistics Bureau (2013) Province of Prince Edward Island Thirty-Ninth Annual Statistical Review, 2012. Department of Finance, Charlottetown

Prince Edward Island Canada (2003a) 2000/02 Prince Edward Island Corporate Land Use Inventory Land Use and Land Cover Summary. Resource Inventory and Modeling Section, Department of Agriculture and Forestry, Charlottetown

Prince Edward Island Canada (2003b) 2000/02 Prince Edward Island Corporate Land Use Inventory Forest Summary: Forestry. Working now for the Future. Resource Inventory and Modelling Section Department of Agriculture, Fisheries, Aquaculture and Forestry, Charlottetown

Prince Edward Island Canada (2003c) State of the Forest Report 1990-2000. Planning for the Future. Department of Agriculture and Forestry Forestry and Land Resource Modelling Division, Chartlottetown

Smith Green & Associates (1995) P.E.I. Wood Fuel Survey: Final report, 1994-95. Dept. of Economic Development & Tourism, Charlottetown

Statistics Canada (1997) Agriculture Census-Recensement, 1996, Agricultural Profile of the Atlantic Provinces, Profil agricole des provinces de l'Atlantique. Minister of Industry, Ottawa

Statistics Canada (1982) 1981 Census of Canada, Recensement du Canada de 1981, Agriculture, Prince Edward Island. Minister of Supply and Services, Ottawa

Tello E, Galán E, Sacristán V, Cunfer G, Guzmán I, González de Molina M, Krausmann F, Gingrich S, Padró R, Marco I, Moreno-Delgado D (2016) Opening the black box of energy throughputs in farm systems: A decomposition analysis between the energy returns to external inputs, internal biomass reuses and total inputs consumed (the Vallès County, Catalonia, c.1860 and 1999). Ecol Econ 121:160–174. doi: 10.1016/j.ecolecon.2015.11.012

2. Data compilation of major energy flows and energy returns

Simone Gingrich, Inés Marco, Eduardo Aguilera, Roc Padró, Claudio Cattaneo, Geoff Cunfer, Gloria Isabel Guzmán Casado, Joshua MacFadyen, Andrew Watson

Table SI1 Major energy flows and energy returns in advanced organic, industrializing and industrialized agroecosystems. Data sources: see text

			Final Produce	External Inputs	Biomass Reused	Total Inputs Consumed	EFEROI	IFEROI	FEROI
		year	GJ/ha/yr	GJ/ha/yr	GJ/ha/yr	GJ/ha/yr			
Europe	St. Florian (AT)	1864	27	3.9	68	71	6.9	0.4	0.4
		1950	20	12	46	58	1.7	0.4	0.3
		2000	109	42	46	88	2.6	2.4	1.2
	Grünburg (AT)	1864	21	1.6	34	36	12.9	0.6	0.6
		1950	14	5.9	44	50	2.4	0.3	0.3
		2000	41	64	67	130	0.7	0.6	0.3
	Vallés (ES)	1860	22	1.0	21	22	21.5	1.0	1.0
		1956	20	7.7	13	20	2.6	1.6	1.0
		1999	34	138	16	153	0.2	2.1	0.2
	Santa Fe (ES)	1904	27	4.6	54	59	5.8	0.5	0.5
		1934	26	5.0	53	58	5.1	0.5	0.4
		1997	81	35	63	98	2.3	1.3	0.8
North America	Queens (CA)	1880	24	2.0	26	28	11.8	0.9	0.8
		1950	24	3.9	31	35	6.1	0.8	0.7
		1995	29	9.3	37	47	3.2	0.8	0.6
	Nemaha (USA)	1880	6	4.6	23	28	1.3	0.3	0.2
		1954	14	3.1	35	38	4.4	0.4	0.4
		1997	32	12	66	78	2.6	0.5	0.4
	Decatur (USA)	1880	0.5	0.4	2.0	2.4	1.2	0.3	0.2
		1954	6.0	1.3	21	23	4.8	0.3	0.3
		1997	17	6.2	31	38	2.7	0.5	0.4

3. Comparative discussion of temporal trends in energy input and output intensity

Simone Gingrich, Inés Marco, Eduardo Aguilera, Roc Padró, Claudio Cattaneo, Geoff Cunfer, Gloria Isabel Guzmán Casado, Joshua MacFadyen, Andrew Watson

Figure SI1 displays the per-area values of Final Produce and different types of inputs. The dots refer to combinations of energy outputs and inputs in pre-industrial (starting point of arrows) and industrialized agroecosystems (end point of arrows), and the direction of arrows corresponds to changes in energy returns per unit of input. This integrates the perspectives of agroecosystem efficiency and intensity (Erb et al. 2013), and combines flow-flow relations, i.e. unit of output per unit of input, with “flow-fund-relations” (Georgescu-Roegen 1971; Giampietro et al. 2009), i.e. unit of energy per unit of area. Figure SI1a compares Final Produce to Total Inputs Consumed and displays that more output was achieved at equally increasing input (all arrows point up and right). It also reveals that, while FEROI values were comparable among case studies, the levels of inputs and outputs diverged significantly between European and North American case studies throughout the time period.

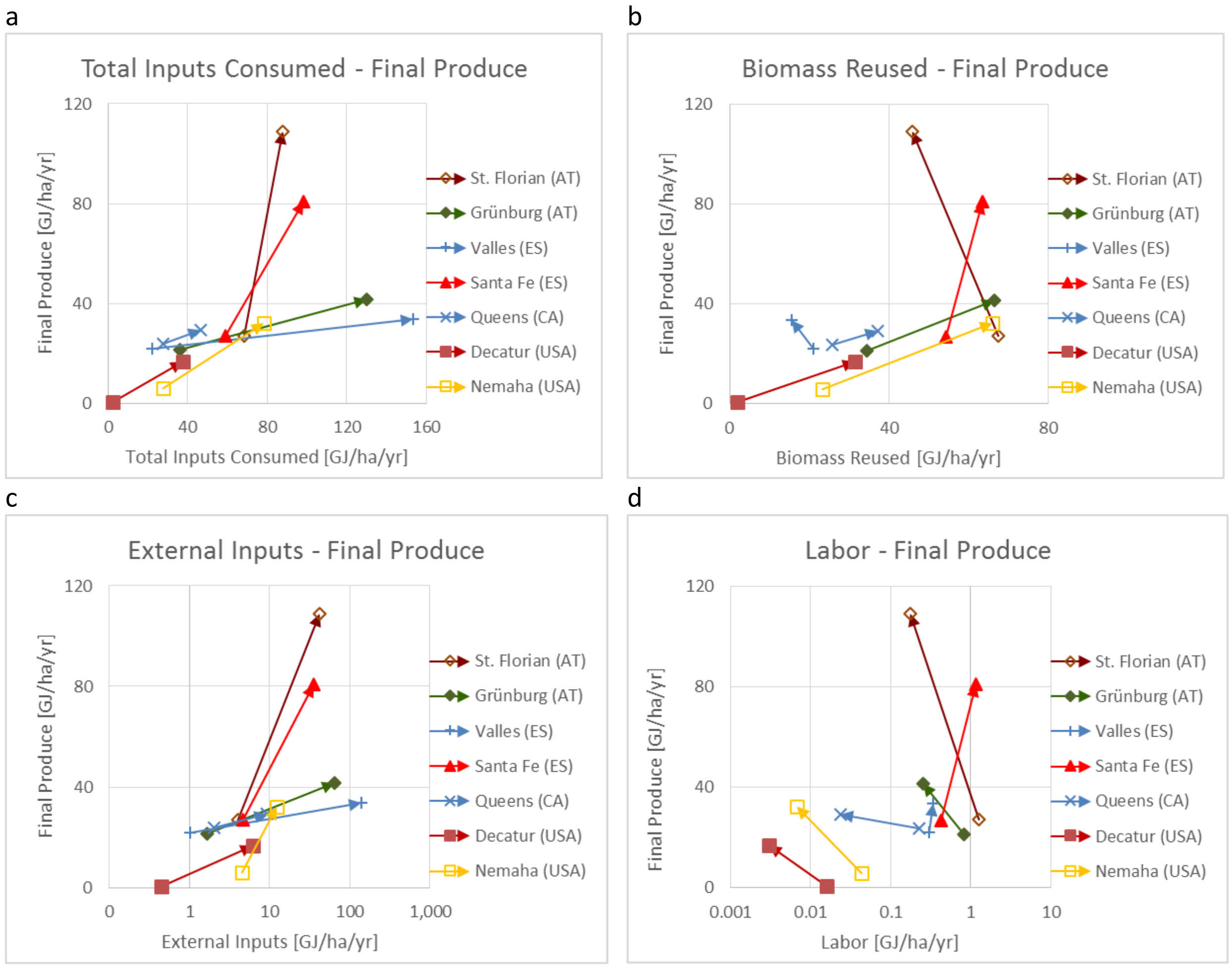

SI Figure 1: area intensity of energy input (a: Total Inputs Consumed; b: Biomass reused; c: External Inputs; d: Labor) and energy output (Final Produce) in advanced organic and industrialized agroecosystems. Arrows point from pre-industrial to industrial values (for time points in the individual case studies, see main text).

Figures SI1b, SI1c and SI1d show that the type of input changed in the course of the agroecosystem energy transition in Western agriculture. The amount of Biomass Reused changed at levels similar to or below those of Final Produce, while external inputs changed much more (arrows point far right on a logarithmic scale). Finally, labor was not only in relative, but also in absolute terms a decreasingly important energy input (arrows point up). It is worth contrasting land productivity (higher in Europe) and labor productivity (higher in North America, see Table 2): In North America, less farmers worked on larger land areas, and generated more Final Produce per unit of energy invested in labor. In combination with differences in biogeographic conditions, the low input of labor in the North American case studies may provide an explanation to the low area productivity values in North America in the late 20th century (Arizpe et al. 2011). Final Produce per unit of labor energy input was between 820 and 2650 (Nemaha and Decatur, respectively), while in Europe, these values ranged between 70 (Santa Fe) and 613 (Sankt Florian).

3.1. References:

Arizpe N, Giampietro M, Ramos-Martin J (2011) Food Security and Fossil Energy Dependence: An International Comparison of the Use of Fossil Energy in Agriculture (1991-2003). Crit Rev Plant Sci 30:45–63. doi: 10.1080/07352689.2011.554352

Erb K-H, Haberl H, Jepsen MR, Kuemmerle T, Lindner M, Müller D, Verburg P, Reenberg A (2013) A conceptual framework for analysing and measuring land-use intensity. Curr Opin Environ Sustain 5:464–470. doi: 10.1016/j.cosust.2013.07.010

Georgescu-Roegen N (1971) The Entropy Law and the Economic Process. Harvard University Press, Cambridge, MA

Giampietro M, Mayumi K, Ramos-Martin J (2009) Multi-scale integrated analysis of societal and ecosystem metabolism (MuSIASEM): Theoretical concepts and basic rationale. Energy 34:313–322. doi: 10.1016/j.energy.2008.07.020
